# Clinical Diagnosis of X-Linked Spondyloepiphyseal Dysplasia Tarda and a Novel Missense Mutation in the Sedlin Gene (SEDL)

**DOI:** 10.1155/2018/8263136

**Published:** 2018-12-10

**Authors:** Lei Kong, Dongxu Wang, Shanshan Li, Chengsheng Zhang, Xiuyun Jiang, Qingbo Guan, Zhenlin Zhang, Fei Jing, Jin Xu

**Affiliations:** ^1^Department of Endocrinology, Shandong Provincial Hospital affiliated to Shandong University, China; ^2^Shandong Clinical Medical Centre of Endocrinology and Metabolism, China; ^3^Institute of Endocrinology and Metabolism, Shandong Academy of Clinical Medicine, China; ^4^Shandong Cancer Hospital, China; ^5^Metabolic Bone Disease and Genetics Research Unit, Department of Osteoporosis and Bone Diseases, Shanghai Jiao Tong University Affiliated Sixth People's Hospital, China; ^6^Shanghai Key Clinical Centre for Metabolic Disease, China

## Abstract

**Objective:**

Spondyloepiphyseal dysplasia tarda (SEDT) is a rare hereditary bone disease characterized by spinal and epiphyseal anomalies. We identified the disease by gene sequencing in a Chinese pedigree with SEDT.

**Methods:**

We extracted genomic DNA from five members of a four-generation Chinese SEDT kindred with three affected males and then analyzed the genetic mutation by PCR and DNA sequencing.

**Results:**

DNA sequencing showed that the genetic missense mutation occurred one bp upstream of exon 6 in the *SEDL* gene in two families, and a heterozygous mutation was found in a female carrier. In addition, no mutation was found in the other members of the family.

**Conclusion:**

SEDT in this family was caused by a G/C missense mutation in exon 6 of the *SEDL* gene, previously not shown to be associated with X-linked SEDT.

## 1. Introduction

The term spondyloepiphyseal dysplasia (SED) encompasses a group of hereditary bone diseases characterized by spinal and skeletal anomalies. On the basis of the clinical features, imaging characteristics and differences in the molecular genetics of SED, the International Classification of Hereditary Osteopathy (2010 edition) classified this kind of hereditary osteopathy into nine types [1]. Among them is X-linked spondyloepiphyseal dysplasia tarda (SEDT) with an incidence of approximately 17 : 1 million [2]. The cause gene *SEDL* is located on Xp22, region 2 of the short arm of the X-chromosome, and encodes the transporter granule complex-2 (transport protein particle complex-2 (TRAPPC2)) [3]. SEDT progresses later than other forms of SED; however, the dwarfism and lumbar vertebrae lesions are characteristic, and the joint abnormalities are relatively mild. SEDT lesions, which occur in childhood, mainly involve the lumbar vertebrae and proximal load-bearing joints leading to a short-trunk type of short stature and early degenerative changes in the joints [4]. Imaging reveals vertebral flatness and epiphyseal dysplasia.

The traditional diagnostic methods of SEDT depend on the typical clinical manifestations and imaging changes in the patients after the onset of the disease [5]. In affected families, a diagnosis can be established by genetic screening of asymptomatic young individuals [6].

## 2. Patients and Methods

The male patient is a 14-year-old student who went to the hospital for growth retardation within the past two years. The parents are not close relatives, and the mother was not exposed to radiation or toxic chemicals during pregnancy. According to the parents' description, the patient was normal in length and weight at birth and was proportionate in stature with no obvious history of chronic organic diseases. Two years ago, it was found that the height growth rate of the patient was slower than that of children of the same age, and the annual growth rate was less than 5 cm. Then, the patient went to a local hospital for consideration of the pectus carinatum and began treatment with calcium and zinc supplementation, although the effect has not been obvious. The physical examination showed that the patient's height was 151 cm, 2 SD lower than the mean of the normal reference value for individuals of the same sex, age, area, and race. The patient's weight was 46 kg, with an upper body measurement of 73 cm, a lower body measurement of 78 cm, and finger spacing at 156 cm. There was no obvious abnormality in facial appearance, no low hairline, and no obvious widening of binocular distance, and the patient's intelligence was average. In terms of sexual characteristics, the patient had grown a small amount of beard, axillary hair, and pubic hair, which reflects male distribution. The scrotum was normal in volume and pigmentation, with a penile length of approximately 10 cm. In addition, cardiopulmonary examination was within the normal limit, spinal flexion was within the normal physiological range, the limb joints were not deformed, limb muscle strength was normal, the hands did not have intersecting palms, and the fourth metacarpal bones were not short.

**Figure 1:**
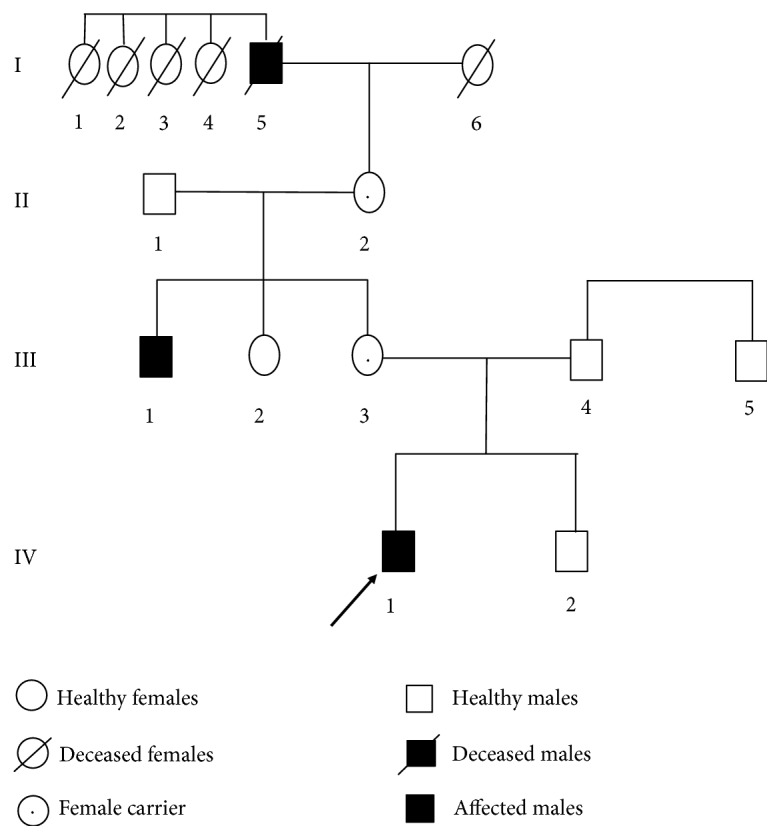
This is the pedigree of a Chinese SEDT family. I, II, III, and IV indicate the first to fourth generations of the families, and the arrow indicates the proband (IV1). All open boxes represent healthy males, and open circles represent healthy females. Filled boxes represent affected males. Boxes or circles with a crossed line indicate that the person has already died. All circles with a dot in the middle indicate the status of the carrier.

**Figure 2:**
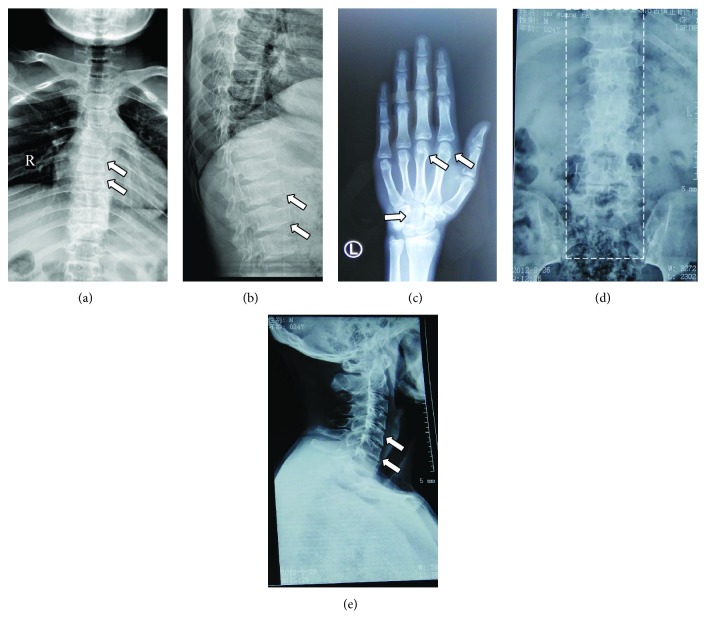
Radiographs of the patients of kindred members. The X-ray findings of the proband were as follows. (a, b) Thoracolumbar anteroposterior position; the arrows indicate anterior superior and inferior edge depression, posterior hump protuberance, and milk bottle sign. (c) Lefthand positive position, interphalangeal joint enlargement as shown by arrow, carpal fusion. (d, e) The X-ray findings of the proband's uncle. (d) Total spinal position, scoliosis. (e) Cervical vertebrae lateral position; the arrows indicate superior and inferior edges of vertebral body depression, milk bottle sign.

The level of pituitary, thyroid, sex, and cortisol hormones as well as the rhythm measurement were normal in the general laboratory examination. Ultrasonic examination of the liver, gall bladder, pancreas, spleen, and kidney showed no obvious abnormalities; the thyroid showed a follicular cyst; and the structure of the heart was generally normal. The peak value of the growth hormone stimulation test was 43.67 ng/ml, and the level of IGF-1 was not exceptional. The number of chromosomes was 46, XY. The evaluation of the growth and development of the patient showed that the bone age of the patient was 15.5 years old, which was younger than his chronological age. The left knee joint metaphysis was not completely closed. The family history was as follows: the father was 175 cm tall, and the mother was 158.5 cm; they are not related, and both are healthy. The patient's brother, who is 9 years old, is 139 cm. The patient's uncle presented with a short trunk (height is 145 cm), short neck, chest deformity, and degenerative joint disease. Moreover, according to the narration of the patient's family member, the patient's uncle's grandfather is also of similar stature. The pedigree was drawn (Figure 1), and the X-ray auxiliary inspection was completed (Figure 2).

### 2.1. Genome Sequencing

#### 2.1.1. DNA Extraction and Quality Examination

Peripheral blood samples were obtained with informed consent from the five individuals (the proband IV1, the brother of the proband IV2, the parents of the proband III3 and III4, and the uncle of the proband III1) and then stored in EDTA anticoagulant tubes. Genomic DNA was extracted from blood samples using a genomic DNA purification kit according to the manufacturer's instructions and was stored at −80°C. A total of 1 *μ*l of each DNA sample was visualized by 1% agarose electrophoresis, and the concentration was estimated. Then, the samples were diluted to working concentrations of 5-10 ng/l according to the estimated concentration, and samples without obvious DNA bands were not diluted.

#### 2.1.2. Polymerase Chain Reaction Amplification and Sequencing

The following reaction conditions were used for PCR: condition one, applied to the six fragments except *SEDL* 2F/R. The reaction mixture (20 *μ*l) included 1x GC buffer I (TAKARA), 2.5 mM Mg2^+^, 0.2 mM dNTPs, 0.2 *μ*M each primer, 1 U of HotStarTaq polymerase (TAKARA), and 1 *μ*l of template DNA. The cycling program included 95°C for 2 min; 11 cycles of 94°C for 20 s, 64°C-0.5°C/cycle for 40 s, and 72°C for 1 min; 24 cycles of 94°C for 20 s, 58°C for 30 s, and 72°C for 1 min; 72°C for 2 min; and a 4°C hold. Condition two, for one fragment of *SEDL* 2F/R. The materials are the same as those mentioned above. The cycling program included 95°C for 2 min; 35 cycles of 96°C for 10 s and 68°C for 1 min; and a 4°C hold.

*(1) PCR Purification Using SAP and Exo I*. To purify the PCR products, 0.5 U of SAP and 4 U of Exo I were added to 8 *μ*l of PCR products. The mixture was incubated at 37°C for 60 min, followed by incubation at 75°C for 15 min (PCR primers, Table 1).

*(2) Sequencing Reaction*. The reaction mixture included 3 *μ*l of BigDye 3.1 mix, 2 *μ*l of sequencing primer (1 *μ*M), and 1-2 *μ*l of purified PCR product. The cycling program used was as follows: 96°C for 1 min; 28 cycles of 96°C for 10 s, 50°C for 5 s, and 60°C for 4 min; and a 4°C hold (sequencing primers, Table 2). An ABI 3730xl sequencer was used to sequence the products, the sequencing files were analyzed by PolyPhred software, and the results were clarified by combining them with records that had been manually proofread.

## 3. Results

### 3.1. Case Analysis

This case is a large pedigree consisting of a four-generation Chinese SEDT kindred with three affected males (the first-generation patient was diagnosed by description but was not confirmed). The patients are all male, which is in accordance with the genetic characteristics of X-linked recessive inheritance, and the imaging features are consistent with the typical characteristic features of SEDT. X-ray examination of the patient in this study showed that the vertebrae were flattened in the middle position (Figure 2(a)); the anterior and lower margins of the vertebrae were sunken in the lateral position, with central and posterior hump-like protuberances, and the vertebrae presented with the appearance of a milk bottle (Figure 2(b)); the palmar metacarpal and phalangeal epiphysis was enlarged. The metacarpophalangeal and interphalangeal joints were enlarged, and the carpal bone was fused (Figure 2(c)). The imaging data of the proband's uncle (III1) showed that the spine was prone to scoliosis. The cervical vertebrae was morphologically altered, showing a milk bottle sign similar to that of the patient (Figures 2(d) and 2(e)).

### 3.2. Analysis of Gene Detection

**Figure 3:**
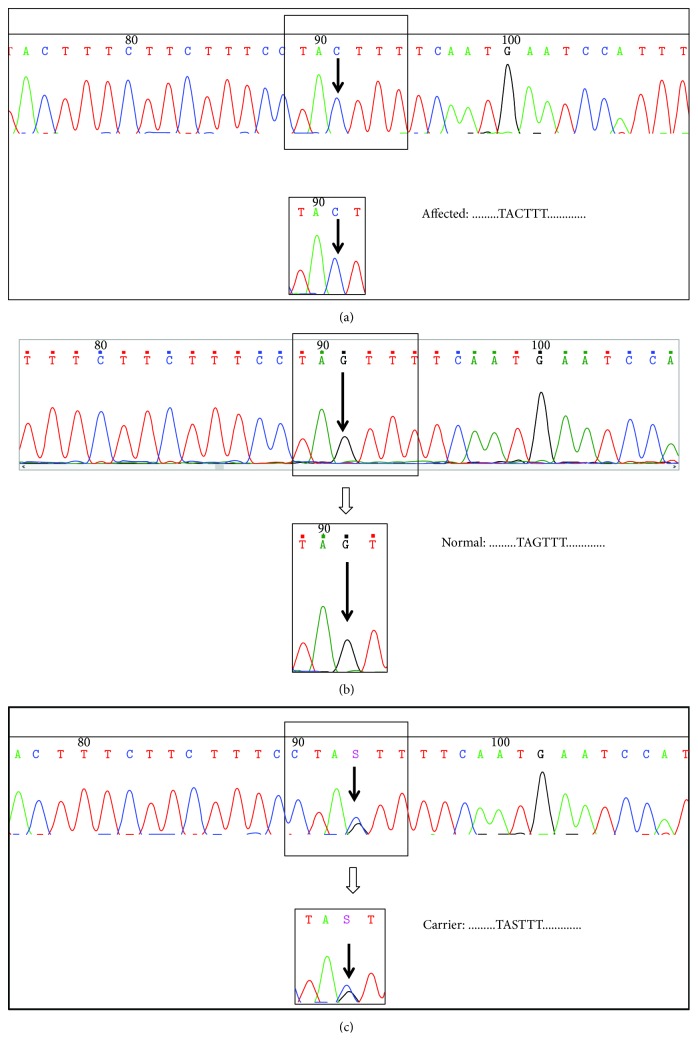
Sequencing results of the *SEDL* gene. As shown in the diagram, panels (a), (b), and (c) represent the patient sequence, the normal sequence, and the carrier sequence, respectively. The location of the arrow is the mutation site reported in the study. (a) Patient sequence (hemizygote), a missense mutation occurred in the patient's gene, G to C. (b) Normal sequence, the normal base in the gene at the sequenced site is C. (c) Carrier sequence (heterozygote). The mutation in female carriers is S (G + C).

All exons of the *SEDL* gene were sequenced from the five samples of the proband and his family. The results showed that the *SEDL* gene in the proband (IV1) was mutated compared with the normal *SEDL* sequence (Figure 3(b)). A mutant site was identified in the proband, which was located 1 bp upstream of exon 6. The missense mutation (homozygous mutation) changed a G into a C, which led to the possible change of a splicing site (Figure 3(a)). *SEDL* gene mutations in two samples were found in relatives of the proband. The mother (III3) of the proband was a carrier, and the missense mutation (heterozygous mutation) occurred 1 bp upstream of exon 6, leading to a possible mutation of the splicing site (NM_001128835.2) (Figure 3(c)). The genotype of the mother changed, although the phenotype had not. The sequencing results of the uncle were consistent with the proband: the missense mutation (homozygous mutation) changed a G to a C 1 bp upstream of exon 6, leading to a possible mutation of the splicing site (NM_001128835.2). The sequencing results of the proband's father (III4) and younger brother (IV2) were normal (Figure 3(b)).

## 4. Discussion

Spondyloepiphyseal dysplasia tarda (SEDT) is an X-linked recessive hereditary disease, which was first reported by Gedeon in 1999 [7]. The gene was highly conserved in the process of evolution, with a total length of 20 kb, containing six exons and encoding the protein Sedlin, which contains 140 amino acid residues [8, 9]. Current research has suggested that the *SEDL* protein affects transport from the endoplasmic reticulum to the Golgi apparatus, resulting in the obstruction of protein transport in cells (especially chondrocytes), which makes it impossible to secrete collagen to form a normal extracellular matrix structure, leading to retardation of chondrocyte development and eventual disease manifestations [8, 10]. At present, 48 *SEDL* gene mutations have been reported (data from HGMD and PubMed searches), including 25 deletion mutations, 13 missense/nonsense mutations, 6 splicing mutations, 2 insertion mutations, and 1 insertion/deletion mutation [11, 12]. In this study, the guanine, G, 1 bp upstream of exon 6 changes to a cytosine, C, which is a missense mutation. So far, there have been no other reports of this mutation. The severity of the disease was not completely uniform among patients with *SEDL*, because different genotypes of *SEDL* have different phenotypes [13]. It is generally believed that the clinical manifestations of mutations near the 3′ end of the *SEDL* gene are relatively mild, while those near the 5′ end are more serious, and the patients with mutations at the 3′ end of exon 4 may have severe hip pain and spinal curvature in the early stages [10].

The typical clinical manifestations of SEDT were spinal and skeletal anomalies. Imaging findings showed that the vertebra presents the appearance of a milk bottle [9, 14]. With increasing age, the progressive changes in the bone are obvious, the large joint is deformed, and findings include scoliosis of the spine and backward protrusions.

Dwarfism is often accompanied by endocrine hormone imbalance during childhood, so clinicians will consult the results of endocrine hormone test in the first. When the endocrine hormone of the patient is normal, the clinician will focus on the physical condition of the patient, and the imaging examination will be carried out. In this case, the two patients in this study showed characteristic imaging changes, but there were no signs of the systemic biochemistry, abnormal hormone metabolism, or unspecific phenotypic changes in the preexamination. Therefore, in order to reduce the misdiagnosis of bone metabolic diseases, the family history should be investigated in detail, the physical examination should be completed, and at the same time, X-ray examination of the skull, pelvis, vertebrae, extremities, and other parts should be implemented. Even so, it is difficult to distinguish SEDT from mucopolysaccharide storage type IV, idiopathic dwarfism, congenital spinal epiphyseal dysplasia, multiple epiphyseal dysplasia, etc., because the clinical features of the disease are not obvious during early childhood [15, 16].

SEDT is a progressive disease, seriously affecting the quality of life of patients, and there is no effective treatment; symptoms are alleviated mostly according to the signs and clinical manifestations of patients, and patients are told to avoid injury to the spinal cord and activities involving the large joints; joint replacement may be considered for severe involvement [17]. Therefore, prevention and early diagnosis are the key points in the control of the disease. Gene diagnosis can be used to diagnose the disease as early as possible and improve the late symptoms of the patients. Meanwhile, it can identify multiple *SEDL* gene mutations and avoid misdiagnosis. However, clinicians sometimes cannot confirm the possible scope of the disease according to their clinical symptoms, and the target sequencing of specific genes often results in negative results. Therefore, it is particularly necessary to do the whole exome sequencing (WES) for rare diseases. Previous studies have shown that the diagnostic rate of WES for undiagnosed diseases is higher than that of classical genetic diagnosis and called for WES as a first-line diagnostic tool for undiagnosed diseases [18]. The presymptom diagnosis of genome screening can help identify the patient so that he or she can obtain reasonable suggestions for his or her lifestyle, and prenatal diagnosis can identify female carriers to provide them with genetic counseling and eugenics and then encourage bearing and rearing better children [19].

## Figures and Tables

**Table 1:** PCR primer sequences of the SEDL gene.

PCR primer	Forward/reverse	Primers (5′-3′)
*SEDL* P1	F	AGGGAACGTGAACGTCTGAAA
	R	TCTTCAGCTCGGGAAGGCTAT
*SEDL* P2	F	CCGAGGGTTCGGGAGGAACAAAG
	R	CAGCGGAGGGCTGGCAGGTC
*SEDL* P3	F	CGGATGTTGGTCCTGTACCTC
	R	ATCAAGGCCCCGATAAAGACA
*SEDL* P4	F	CATGAGAATGTTGTCTTTGTGATTTC
	R	CGGCAATCCACTACAGGTGA
*SEDL* P5	F	TCGGTAACTTCCTTCTTGAAACATGA
	R	AAATCACCCTTGATAGGGTCCA
*SEDL* P6	F	TCTGGACCCTATCAAGGGTGA
	R	CCCTAATAAAATCAGCTATGAGGACA
*SEDL* P7	F	AATGTGGTCTTTAGACTTTGGAATG
	R	CAAAAGTTTTCCAGGCTATTTAATCA

**Table 2:** Sequences of sequencing primer.

PCR primer	Forward/reverse	Primers (5′-3′)
*SEDL* P1	F	AGGGAACGTGAACGTCTGAAA
*SEDL* P2	F	CCGAGGGTTCGGGAGGAACAAAG
*SEDL* P3	R	ATCAAGGCCCCGATAAAGACA
*SEDL* P4	F	CATGAGAATGTTGTCTTTGTGATTTC
*SEDL* P5	F	TCGGTAACTTCCTTCTTGAAACATGA
*SEDL* P6	F	TCTGGACCCTATCAAGGGTGA
*SEDL* P7	F	AATGTGGTCTTTAGACTTTGGAATG

## Data Availability

The data used to support the findings of this study are available from the corresponding author upon request.
